# Transcriptome and Metabolome Analyses Uncover Genes and Pathways Linking Growth Trajectories to Cardiometabolic Risk Markers in Childhood

**DOI:** 10.3390/cimb48020238

**Published:** 2026-02-23

**Authors:** Reena Perchard, Terence Garner, Lucy E. Higgins, Philip G. Murray, Amirul Roslan, Edward D. Johnstone, Adam Stevens, Peter E. Clayton

**Affiliations:** 1 Division of Developmental Biology & Medicine, Faculty of Biology, Medicine & Health, University of Manchester, Manchester M13 9WL, UK; terence.garner@manchester.ac.uk (T.G.); lucy.higgins@manchester.ac.uk (L.E.H.); amirul.roslan@manchester.ac.uk (A.R.); edward.johnstone@mft.nhs.uk (E.D.J.); adam.stevens@manchester.ac.uk (A.S.); peter.clayton@manchester.ac.uk (P.E.C.); 2 Manchester University NHS Foundation Trust, Manchester M13 9WL, UK; philip.murray@mft.nhs.uk

**Keywords:** fetal growth restriction, cardiometabolic risk, transcriptomics, metabolomics

## Abstract

Small for gestational age (SGA) is often used as a proxy for fetal growth restriction (FGR), yet not all FGR fetuses are born SGA. SGA individuals, particularly those with catch-up growth, have increased cardiometabolic risk. We therefore studied infants and children from pregnancies at increased FGR risk, irrespective of birthweight. Two cohorts enriched for suboptimal fetal growth were recruited: an infant cohort (N = 80) to examine relationships between fetal weight trajectory and postnatal growth and a cohort of children aged 3–7 years (N = 80), 31 of whom provided blood samples for transcriptome and metabolome analyses. In infants, fetal weight trajectory correlated negatively with BMI change from birth to three months (R = −0.40, *p* = 0.004) and six months (R = −0.38, *p* = 0.012), as well as with skinfold, abdominal and arm circumferences. In children, supervised transcriptome analysis highlighted a pathway including *ARG1*. Unsupervised analysis had previously identified two SBP-differentiated groups; novel findings include *LATS1,* implicated in SBP GWAS, as the most significant gene, and *GHRL*, suggesting appetite-regulation mechanisms underlie SBP differences. Ornithine, a differentially expressed metabolite between fetal and childhood weight trajectory quartiles, together with *ARG1,* suggested involvement of the arginine-nitric oxide pathway. Early life indicators of cardiometabolic risk have been elucidated, highlighting pathways to inform future prevention.

## 1. Introduction

The fetal origins hypothesis [1] states that fetal undernutrition leads to metabolic adaptations that persist throughout life. In the presence of plentiful resources postnatally, these adaptations confer a disadvantage to the exposed individual. Whilst the original focus was on long-term consequences of low birthweight, this hypothesis has provided reason to explore the programming of health and disease.

Studies that examine the links between low birthweight (LBW) or small-for-gestational age (SGA, birthweight < −2 SDS) and childhood markers of cardiometabolic risk use SGA as a surrogate marker for fetal growth restriction (FGR). However, many individuals may be exposed to suboptimal fetal growth (SFG) without being born SGA [2,3,4]. Therefore, we have focused on studying children born following pregnancies at greater risk of FGR.

Previous work evaluated cardiometabolic risk profiles in the Avon Longitudinal Study of Parents and Children (ALSPAC) cohort and assessed whether mid-childhood transcriptomic profiles could predict later risk, validating findings in a cohort of three- to seven-year-old children, enriched for those who had been born following pregnancies with SFG [5]). For the current work, to characterise fetal and postnatal growth trajectories in early life following pregnancies with SFG, two cohorts of children enriched for SFG have been studied; the first recruited from a specific antenatal clinic with postnatal follow-up to 12 months, with the second being the three- to seven-year-olds (whose mothers had been seen in that same clinic) used to validate the ALSPAC findings. Now, these cohorts have been used (i) to identify relationships between fetal weight trajectories, weight/adiposity trajectories in early and later childhood, and (ii) to elucidate key genes and metabolic pathways associated with these trajectories and with systolic blood pressure (SBP) as a marker of cardiometabolic risk.

## 2. Materials and Methods

### 2.1. Approvals

Ethical approval (REC reference 17/NW/0153, IRAS ID 187679) and Health Research Authority (HRA) approval were obtained for the Manchester BabyGRO Study. The study was adopted onto the NIHR Clinical Research Network Portfolio.

### 2.2. Recruitment

Participants were recruited into two cohorts between May 2017 and October 2018, with the aim of recruiting 80 participants for each cohort.

### 2.3. Antenatal Recruitment of Pregnant Women and Their Infants

Pregnant women were antenatally recruited to study associations between prenatal and postnatal growth trajectories in their offspring. Women with abnormal serum markers of placental function, i.e., low pregnancy-associated plasma protein A (PAPP-A) or raised Inhibin/alpha-fetoprotein (AFP), indicating greater risk of FGR, were recruited from the Manchester Placenta Clinic (MPC) at St. Mary’s Hospital, Manchester Foundation Trust. This is a tertiary clinical service established in April 2009 to provide midwifery and Obstetric care to women who are pregnant with or are at risk of a growth-restricted fetus. For these women, at 23 weeks’ gestation, measurements of placental size and uterine artery Doppler (UtAD) impedance are taken to classify pregnancies as having higher or lower risk for severe FGR. Factors contributing to higher FGR risk are UtAD pulsatility index (PI) > 1.3 (>95th centile) ± resistance index (RI) > 0.7 (95th centile) ± presence of notching, placental diameter < 10 cm (25th centile), and greater placental depth.

Clinic lists were screened weekly and pregnant women with low PAPP-A (<0.415 MoM), high AFP (>2.2 MoM) or raised inhibin (>2 MoM) were identified. They were approached and informed of the study at 23 weeks, then re-approached for consent at the next clinic visit for participation of their offspring.

### 2.4. Recruitment of Children Aged Three to Seven Years

A recruitment pool (N = 226) was formed of children aged between three and seven years, whose mothers had been seen in the MPC, where the pregnancy had resulted in a livebirth above 34 weeks. To extend the range of fetal weight trajectories included, healthy children were also recruited as offspring of women with no history of pregnancy complications [5,6]. Characteristics of this cohort have been previously published [5].

### 2.5. Exclusion Criteria

Exclusion criteria were a lack of understanding despite the offer of an interpreter, maternal age below 16 years and detection of a congenital anomaly. Any child with a medical condition potentially affecting growth or cardiometabolic risk was also excluded.

## 3. Measurements

### 3.1. Infants

Auxological measurements were collected at birth and at three, six and twelve months of age. All measurements were taken three times and the mean was calculated. Crown-rump and crown-heel lengths were measured using a Seca 417 measuring board and recorded to the nearest 0.1 cm. Weight was measured with a Weylux (BMI 200, Lancashire, UK) digital scale and recorded to the nearest gram. Mid-upper arm (MUAC), abdominal (AC), thigh (TC) and head circumferences (HC) were measured using a standard tape measure and recorded to the nearest 0.1 cm. Biceps, triceps and subscapular skinfold thicknesses were measured using Holtain callipers, recorded to the nearest 0.2 mm, and the sum of skinfold thicknesses (sum SF) was calculated.

### 3.2. Children: Clinical Measures of Cardiometabolic Risk

As previously described [5], height was measured using a Harpenden stadiometer and recorded to the nearest 0.1 cm. Body mass index (BMI) was calculated as weight (in kilograms) divided by height (in metres) squared. MUAC, AC, TC and HC were recorded to the nearest 0.1 cm. Biceps, triceps, subscapular, suprailiac and abdominal skinfold thicknesses were measured using Holtain callipers, recorded to the nearest 0.2 mm, and the sum of skinfolds calculated.

Body composition was measured by air displacement plethysmography (BODPOD, Cosmed^®^, Concord, CA, USA). This [7] follows a similar principle to hydrostatic weighing, the gold standard for body composition assessment. Fat mass (grams), fat mass percentage (%), fat-free mass (grams) and fat-free mass percentage (%) were recorded.

Aortic pulse wave velocity, a non-invasive measure of aortic wall stiffness [8] was performed using the Tensiomed Arteriograph (Tensiomed^®^, Budapest, Hungary). Brachial augmentation index (a measure of the strength of the wave reflection, calculated as the ratio of the amplitude of the reflected wave), aortic pulse pressure, SBP, diastolic blood pressure (DBP), pulse pressure, mean arterial pressure (MAP, all mmHg) and heart rate were also recorded.

### 3.3. Children—Serological Measures of Cardiometabolic Risk

Fasted peripheral blood samples were collected for measurements of glucose, insulin, cholesterol, high-density lipoprotein (HDL), low-density lipoprotein (LDL), triglycerides, IGF-I, cholesterol, and total cholesterol: HDL ratio and non-HDL cholesterol. These were measured using clinical-grade assays.

### 3.4. Children—Transcriptomics and Metabolomics

A total of 2.5 mls of blood was collected and stored at −80 °C in a PAXgene^®^ collection tube for immediate stabilisation of RNA. The remaining blood was transferred into a 4 mL serum vacuette tube, centrifuged at 3000 rpm for 10 min at 4 °C, then stored at −80 °C. 0.5 mL aliquots of stored serum were transported to an external laboratory for metabolomic analysis.

### 3.5. Statistical Analyses

**Figure 1:**
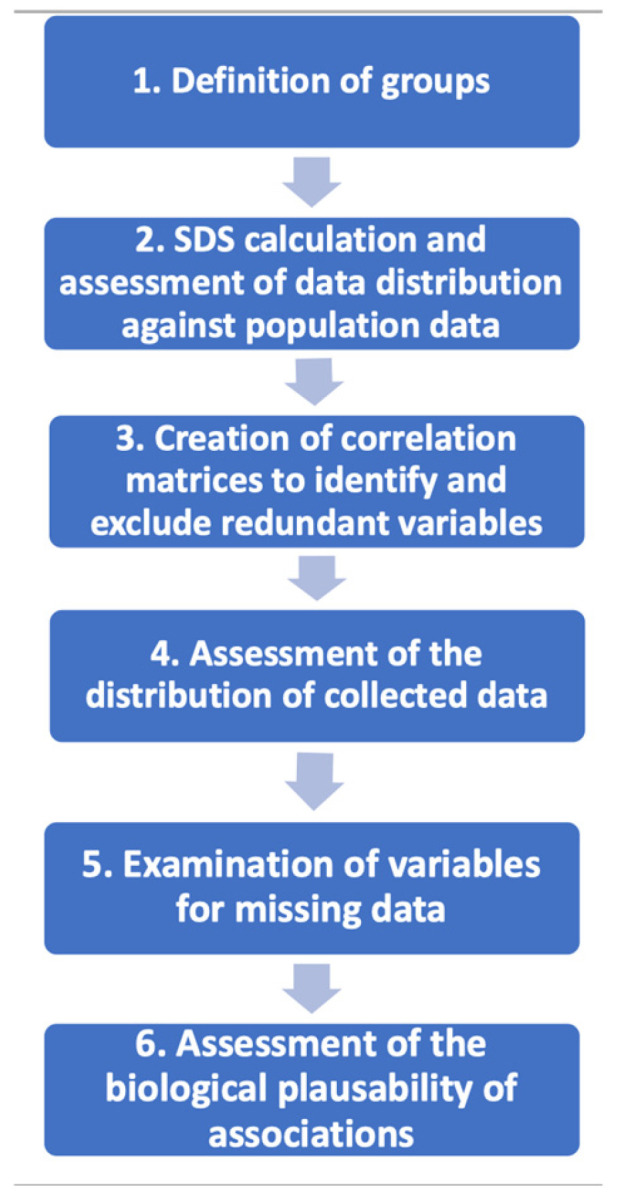
Data curation methods. First, groups were defined, including markers of glucose metabolism, lipid metabolism, and vascular health. These groups helped to inform which representative variables to retain downstream in the curation process. Second, standard deviation scores (based on World Health Organisation data) were calculated, allowing assessment against population data. Third, a correlation matrix was established to determine which variables were highly correlated, thereby aiding the selection of representative variables. Fourth, for each variable, data distribution was assessed to examine whether a transformation may be required in further analyses. Next, the data were examined for missingness, and variables with a high proportion of missing data were excluded. Lastly, the biological plausibility of potential associations with long-term cardiometabolic health was assessed.

Data curation methods were undertaken to select variables for analyses (Figure 1).

### 3.6. Infants

Pearson product-moment correlation coefficient (parametric) and Kendall’s tau (non-parametric) were used to test correlations between fetal weight trajectory ((birthweight centile minus 23 week estimated fetal weight centile)/days) and Δ weight SDS as well as Δ adiposity (BMI, AC, TC, MUAC, biceps, triceps and subscapular SF) at birth, three, six and twelve months, divided by age in days between birth and three, six and twelve months. R or tau values were reported to indicate the strength and direction of associations. *p* < 0.05 was considered significant, and *p* < 0.1 was potentially significant.

### 3.7. Children

Correlations were tested using the same statistical methods as for the infants (described above).

### 3.8. Omic Analyses

Standardised changes in weight; ∆fetal wt ((birthweight centile minus 23 week centile estimated fetal weight)/days) and ∆child wt ((weight SDS minus birthweight SDS)/years) were divided into quartiles. Previously, *t*-tests and Mann–Whitney U tests had examined cardiometabolic differences between ∆fetal wt and ∆child wt quartiles [5]. Where cardiometabolic differences were identified, differentially expressed genes (DEGs) and metabolites (DEMs) were established. Alongside this, for transcriptomics data, rank regression was performed with ∆fetal wt and then ∆child wt as the dependent variable. This allowed the most significant genes to be established. Assessment for commonality was undertaken and overlapping genes were used in gene set enrichment analysis (GSEA).

As an alternative approach [5], we used k-means clustering to separate participants into two groups based on the transcriptome. DEGs between groups were determined and GSEA was performed.

## 4. Results

### 4.1. Infants

#### Recruitment

**Figure 2:**
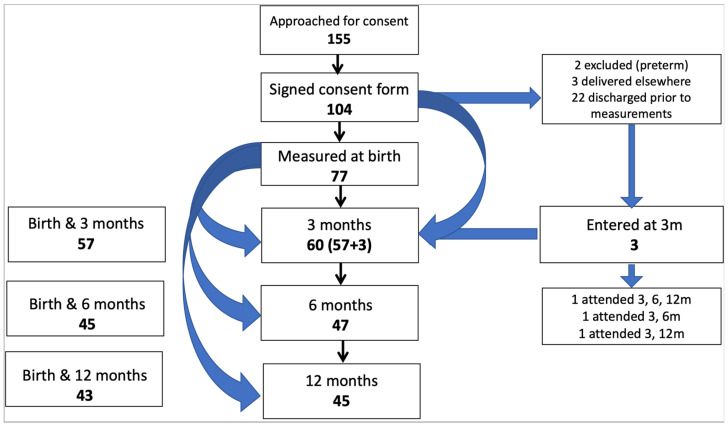
Consort diagram for infant cohort. Of 60 infants measured at three months postnatal age, 57 had also had birth measurements (in addition to birthweight). Of 47 measured at six months, 45 had been measured at birth, and of 45 measured at twelve months, 43 had been measured at birth.

Of 155 pregnant women approached for consent, 104 agreed to participate (Figure 2). In total, 77 babies had measurements at birth, 60 infants were measured at three months, 47 at six months and 45 at twelve months; 34 infants had measurements at all four timepoints.

### 4.2. Baseline Characteristics

The mean (SD) gestational age at birth was 270 (12) days. The mean birthweight SDS of offspring was −0.94. In total, 45 infants were male and 35 were female.

In total, 10% (8/80) were born SGA, defined as birthweight <2nd centile on standardised WHO charts. Using alternative definitions of SGA, birthweight <5th and <10th standardised WHO centiles, SGA rates were 21% and 36%, respectively.

### 4.3. Relationships Between Fetal Weight Trajectory and Postnatal Weight Trajectory

**Figure 3:**
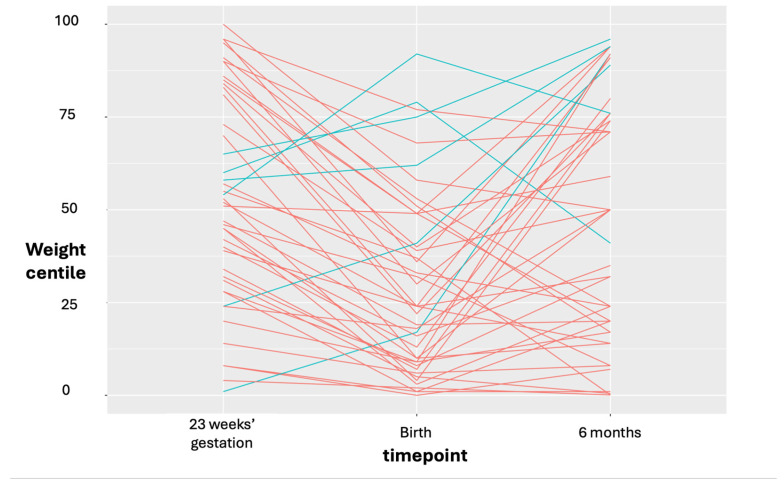
Weight trajectories. The weight trajectories of individual participants between 23 week estimated fetal weight, birth and six months postnatal are shown. Six had a positive fetal weight trajectory, i.e., crossed the centiles upwards (shown in blue), and the rest had a negative fetal weight trajectory (red).

The majority of pregnancies had some degree of SFG (Figure 3).

There were no significant correlations between fetal weight trajectory and Δ weight SDS at birth to three months/age (R = −0.15, *p* = 0.272, N = 58), at birth to six months/age (R = −0.11, *p* = 0.484, N = 46) nor at birth to 12 months/age (tau = −0.10, *p* = 0.398, N = 44).

### 4.4. Fetal Weight Trajectory Correlated Negatively with Δ 0–3 m BMI and with Δ 0–6 m BMI, Sum SF, AC and MUAC

**Figure 4:**
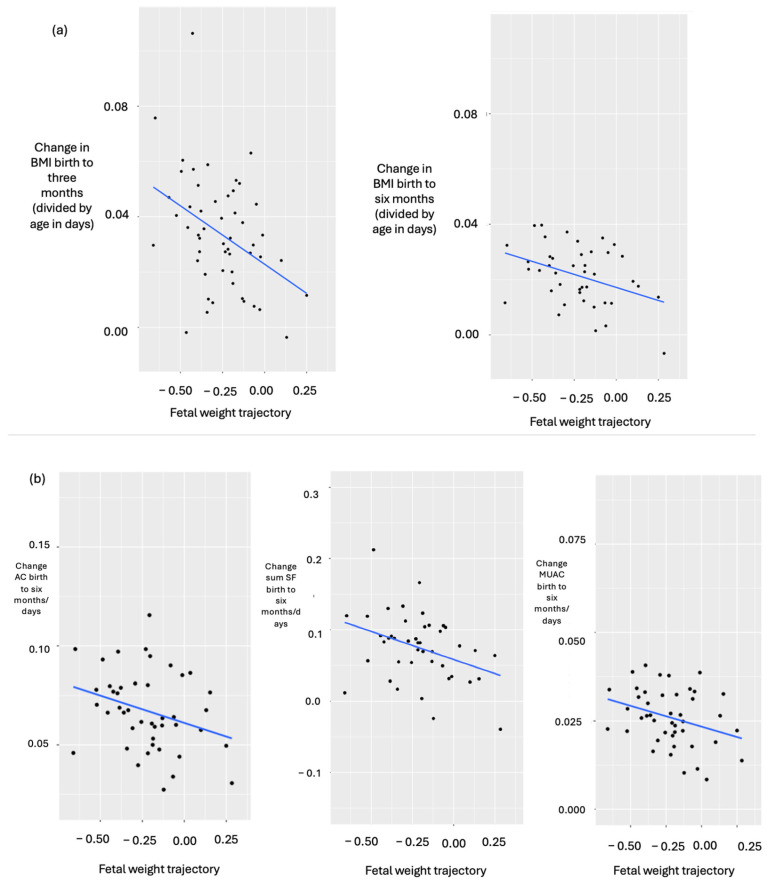
(**a**,**b**). Correlations with fetal weight trajectory in infants. (**a**) Fetal weight trajectory correlated with change in BMI from birth to three months and also from birth to six months. (**b**) Fetal weight trajectory correlated with change in abdominal circumference, change in sum of skinfold thicknesses and change in mid-upper arm circumference from birth to six months.

Fetal weight trajectory correlated negatively with Δ BMI birth to three months/age (R = −0.40, *p* = 0.004, N = 52) and birth to six months/age (R = −0.38, *p* = 0.012, N = 42, Figure 4a), as well as with change in sum of skinfolds (Δ sum SF) birth to six months/age (R = −0.36, *p* = 0.016, N = 43), Δ AC birth to six months/age (R = −0.30, *p* = 0.045, N = 44) and Δ MUAC birth to six months/age (R = −0.32, *p* = 0.034, N = 44, Figure 4b).

### 4.5. Children Aged Three to Seven Years

**Figure 5:**
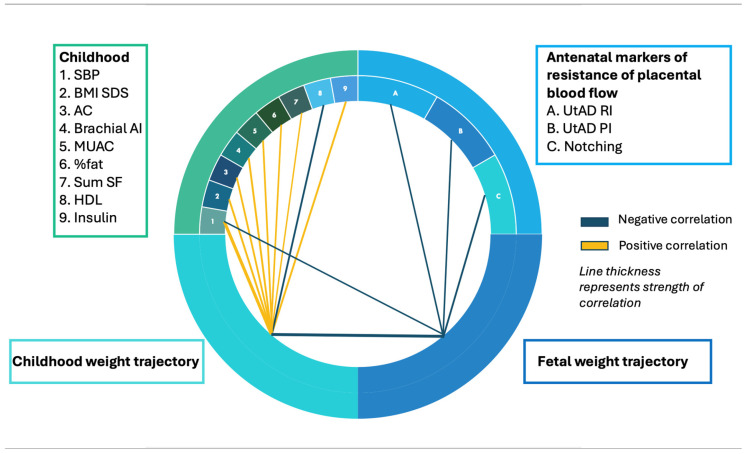
Circos Plot. A number of correlations were demonstrated between antenatal markers, fetal and childhood weight trajectories and childhood markers of cardiometabolic risk. Orange lines represent positive correlations and blue represent negative, with the strongest correlations represented by the thickest lines.

Correlations between ∆fetal wt, ∆child wt and childhood markers of cardiometabolic risk have been previously reported [5] in these 80 children, with SBP showing the strongest associations with both ∆fetal wt and ∆child wt. These findings are summarised in Figure 5 (circos plot). Fetal weight trajectory correlated negatively with UtAD PI, RI and notching; however, no associations were observed with placental diameter, width or depth.

Regression analysis was undertaken to control for potential confounders. Height did not correlate with SBP and was therefore not included. Childhood SBP remained a significant predictor of log_10_(SBP) (β = 0.083, 95% CI 0.036–0.131, *p* = 0.001; model 0.32), whereas birthweight SDS (*p* = 0.45), age (*p* = 0.16), sex (*p* = 0.98), and ethnicity (*p* = 0.31) were not.

### 4.6. Omic Analyses to Identify Pathways

Metabolomic data were available for 26 participants, and transcriptomic data were available for 31. Quality control measures were undertaken for transcriptomic and metabolomic data and cut-offs were defined for quartiles of fetal and childhood weight trajectories.

### 4.7. Transcriptomic Analysis

A rank regression with fetal weight trajectory as the dependent variable and all genes as independent variables found 437 significant genes (unadjusted *p*-value range 1.57 × 10^−4^ to 0.049), and with childhood weight trajectory as the dependent variable, 680 significant genes were identified (unadjusted *p*-value range 1.10 × 10^−4^ to 0.049). Our aim was to prioritise genes with the largest effect sizes rather than those passing multiple correction testing, so we reported unadjusted *p*-values only.

For fetal weight trajectory, assessment of DEGs that were common to results from analysing Q4 versus Q1 of fetal weight trajectory, and rank regression with fetal weight trajectory as the dependent variable revealed 149 genes.

GSEA of the 149 DEGs from both analyses identified groups of genes that share common biological functions and thereby suggested five pathways. These were regulation of trans-synaptic signalling, axon development, neuron projection development, regulation of cytokine production and cellular-modified amino acid metabolic processes. Calculation of normalised enrichment scores allowed quantification of the degree to which each particular set of genes is overrepresented within this set of 149 DEGs.

A total of 193 DEGs were common across all supervised analyses for childhood weight trajectory. Following GSEA for childhood weight trajectory, response to steroid hormone was implicated as the most significant pathway. Genes that accounted for this included *ANXA3* (encoding annexin A3, which cleaves inositol-1,2-cyclic phosphate) and *ARG1* (1.2 times less expressed in the lowest three quartiles relative to the top quartile), encoding arginase, which catalyses the hydrolysis of arginine to ornithine, as well as *DUSP1*, *FOS*, *NR4A1* AND *RBFOX2*. *ARG1* is a steroid-responsive gene regulated by glucocorticoids and androgens through distinct receptor-mediated pathways [9,10] and genetic variation in *ARG1* can influence clinical response to steroid therapy in diseases such as asthma [11,12].

### 4.8. PCA and k-Means Clustering

**Figure 6:**
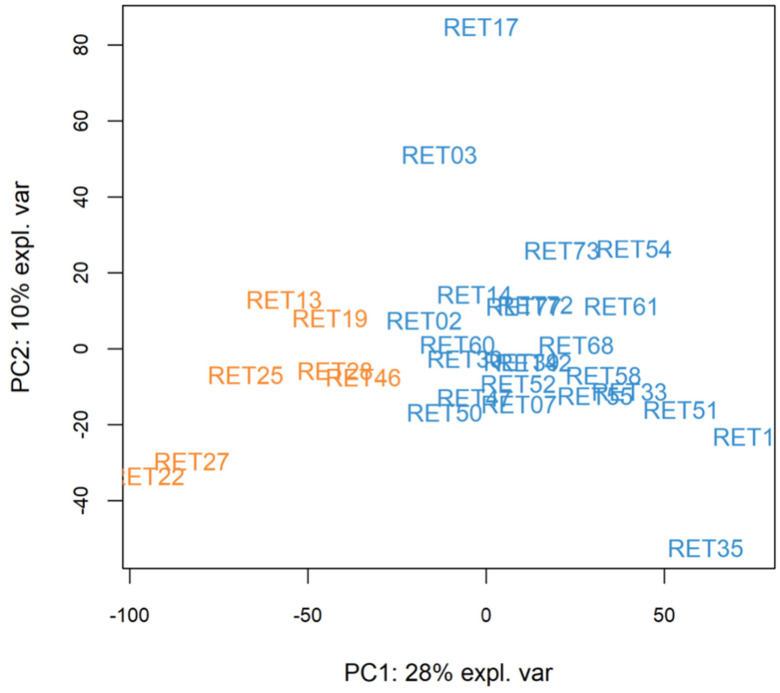
Transcriptomics principal component analysis and k-means clustering. A principal component biplot of the 31 participants for whom transcriptomic data were available. The x and y axes represent the first and second principal components, respectively, which collectively account for the majority of variation in the data. A mathematical algorithm (k-means clustering) was applied to form two separate clusters of participants, based on their transcriptome. The number of clusters was pre-specified as two and the method was otherwise unsupervised. Using this method, each observation was partitioned into a cluster with the nearest mean, the cluster centroid. Within this two-dimensional biplot, k-means clustering used Euclidean distances, which are based on Pythagorean theorem. One cluster included seven participants (orange) and the other included 24 (blue), representing participants at opposite ends of the spectrum.

Unsupervised PCA and k-means clustering had previously identified two groups that differed in SBP [5] (Figure 6).

To assess the stability of the k-means-derived subgroups, we generated silhouette plots. Silhouette coefficients quantify how well each sample fits with its assigned cluster relative to neighbouring clusters, with positive values indicating appropriate cluster assignment. In this dataset, specifying two clusters produced uniformly positive silhouette values (cluster 1: 0.025–0.319, cluster 2: 0.075 to 0.399), indicating that all samples were better matched to their assigned cluster than to alternative groupings. Although these coefficients were modest, consistent with the small sample size, they nonetheless supported the appropriateness of a two-cluster solution. When three, four or five clusters were specified, several samples exhibited negative silhouette values, suggesting poorer separation. Clustering stability was also assessed by subsampling 85% of the participants 200 times (without replacement) and membership compared with that of the full data by Jaccard similarity. Both clusters were considered stable with an average similarity above 80%.

*LATS1* was identified as the most significant DEG between groups (1.14-fold more expressed in the group of 24 relative to the group of 7). *LATS1* encodes an enzyme that regulates cell proliferation and has been previously identified in an SBP GWAS. To further understand differences between the two groups, GSEA based on the most significant genes was performed, and *GHRL* encoding the ghrelin-obestatin prepropeptide was elucidated (0.36-fold less expressed in the group of 24 relative to the group of 7). This suggested a potential role for appetite regulation in differentiating between the two groups that differed in SBP.

### 4.9. Metabolomic Analysis

Rank regression, with fetal weight trajectory as the dependent and all metabolites as independent variables, identified 39 significant metabolites (unadjusted *p*-value range 3.15 × 10^−3^ to 0.049). Rank regression, with childhood weight trajectory as the dependent and all metabolites as independent variables, identified three significant variables (unadjusted *p*-value range from 0.03 to 0.04). There were no significant differences in childhood measures of cardiometabolic risk between the two groups defined by PCA and k-means clustering. Ornithine was the single metabolite identified across all methods.

## 5. Discussion

Having recruited two cohorts whose mothers were followed up in the Manchester Placenta Clinic due to greater FGR risk, enriched for pregnancies with SFG and carefully assessing the characteristics of infants and children born following those pregnancies, relationships have been identified between fetal and postnatal weight trajectories in the first year and fetal and childhood weight trajectories up to mid-childhood. Examining both the transcriptome and the metabolome in three- to seven-year-old children has provided deeper insights into the genes and pathways responsible for later-life cardiometabolic risk development.

### 5.1. Infants

Large cohort studies examining the origins of health and disease have followed subjects from the fetus onwards. Whilst gathering vast amounts of data prospectively, they have not selectively recruited individuals born following greater FGR risk [13,14,15,16,17]. Using unique selection criteria within the BabyGRO study, gains in adiposity between birth and six months but not between birth and twelve months were found to be greatest in individuals who had the lowest intrauterine weight gain. The absence of findings between birth and 12 months may be explained by the small sample size. However, other studies report similar findings in early infancy. As an example, Breij et al. [18] used air displacement plethysmography to longitudinally assess body composition in 203 term infants. The first three postnatal months were identified as a critical window of adiposity development, with a significant increase in % fat (*p* < 0.001) observed. Another study of 401 healthy term infants showed that % fat (measured by the same method) from one to six months but not from six to 12 months was linked with adiposity at two years of age [19]. Together, these studies suggest that adiposity programming, which tracks beyond infancy, occurs by six months of age. Alternatively, the lack of association between fetal and birth to 12-month weight trajectories may reflect the introduction of solid foods by 6 months, with diet acting as a confounder.

### 5.2. Children

There is a clear link between LBW and higher adult BP [20], but evidence suggests that postnatal growth tempo and BMI may be more influential [21,22,23,24,25]. In our small cohort, the strongest association was between childhood weight trajectory and SBP, previously reported in larger studies in SGA [15] which likely reflects the advantage of our unique selection criteria. Other studies performed in the general population have assessed the magnitude of this association. In a study of six-year-old (N = 10,495) children by Tilling et al. [25], all with birthweights above 2500 g, change in SBP per z-score increase in weight gain at 12–60 months was 0.82 mmHg (95% CI 0.58 to 1.06) for boys and 0.97 (0.71 to 1.24) for girls. In the Southampton Women’s study [24], for each SD increase in weight between 12 and 24 months, SBP was higher by 1–2 mmHg (and DBP by 1 mmHg). In our much smaller cohort enriched for pregnancies with SFG, SBP was higher by 3.9 mmHg for each SD increase in childhood weight.

Although BP differences appear modest, a minor SBP reduction could result in a substantial decrease in cardiometabolic risk; lowering SBP in adults by 2 mmHg achieves an 8% decrease in stroke risk [26,27]. Therefore, further studies recruiting individuals following SFG could potentially lead to the early detection of cardiometabolic risk development.

### 5.3. Genes and Pathways Identified

Omic analyses were restricted to those trajectory groups in which cardiometabolic differences had previously been identified [5], enabling a more targeted exploration of underlying biological pathways. Although the sample sizes were small and unadjusted *p*-values are reported, necessitating cautious interpretation, the patterns observed offer potentially important insights. The implications and possible interpretations of these findings are considered below.

L-arginine, a product of the urea cycle, is an immediate precursor of nitric oxide (NO). NO synthesis is catalysed by endothelial nitric oxide synthase (eNOS) enzymes [28], which are expressed in vascular endothelial cells. The role of NO as a powerful vasodilator is well recognised [29,30]. It also plays an important role in insulin signalling. Specifically, NO potentiates glucose-stimulated insulin release mediated by calcium release from endoplasmic reticulum and mitochondria. Additionally, it increases dissociation of glucokinase from insulin secretory granules, thereby augmenting insulin secretion [31].

NO has value as an early marker for human hypertension; impaired NO release acts as a marker of endothelial dysfunction, which is seen early in hypertension development. Studies have also investigated the role of NO as a neurotransmitter [32], modulating BP through sympathetic nervous system activity. This could be important [33] since sympathetic overactivity has been implicated in the early stages of human hypertension [34]. Hypertension has been reported in urea cycle disorders [35], although central nervous system manifestations (altered level of consciousness, seizures) resulting from hyperammonaemia are the major consequences.

Direct relationships between plasma concentration of l-arginine and measures of vasodilation have been demonstrated [36]. In a small study, eight healthy adult male participants who received 30 g IV l-arginine exhibited lower BP (mean 4.4 +/− SE 1.4%) and peripheral vascular resistance (10.4 +/− 3.6). Peripheral vascular resistance was calculated as 80 × (mean BP)/cardiac output. Urinary nitrate excretion rate, an acceptable measure of NO formation in humans [37,38], was measured. A close linear relationship with l-arginine level was found, in line with previous findings [39]. Collectively, these results support that exogenous l-arginine could induce vasodilation through increased production of endogenous NO.

Following assessment for commonality between supervised analyses for childhood weight trajectory and subsequent GSEA, the most significant pathway identified was the response to steroid hormones. Of note, one of the six significant genes involved in this pathway was *ARG1*. *ARG1* encodes arginase, the enzyme that catalyses the hydrolysis of l-arginine to l-ornithine and urea. Therefore, this finding may represent a link between the results of metabolomic and transcriptomic analyses. Furthermore, there is evidence that upregulation of arginase inhibits eNOS-mediated NO synthesis [40]. This upregulation has been implicated in the pathogenesis (endothelial dysfunction) seen in hypertension, diabetes and ageing.

Another gene implicated in this pathway was *ANXA3*, encoding Annexin A3, an enzyme that cleaves inositol 1,2-cyclic phosphate (a derivative of 1-D myoinositol). This results in the formation of inositol 1-phosphate [41]. Phospholipids have been shown to have potential value for the detection of the SGA neonate in early pregnancy. Furthermore, differences in urinary myoinositol have been detected between SGA and appropriate-for-gestational-age neonates at birth [42], as well as between SGA catch-up and non-catch-up [43]. Importantly, inositol derivatives are associated with insulin resistance [44]. In our cohort, there was a trend towards higher insulin for children in the highest quartile of childhood weight trajectory compared with the lowest (Q4 mean 29.9 pmol/L (SD 13.1) versus Q1 20.5 (10.5), *p* = 0.085). It is possible that a difference exists and that our study was underpowered to detect this.

K-means clustering is an established method for clustering large datasets and has been used in previous studies assessing fetal growth [45] and BP variability [46]. In our study, this approach resulted in the formation of two clusters that significantly differed in SBP [5]. The most significant DEG between clusters formed by k-means was *LATS1*. *LATS1* encodes large tumour suppressor kinase 1, an enzyme involved in the Hippo signalling pathway and regulation of cell proliferation. As a tumour suppressor gene, it has also been shown to be downregulated in some cancers, such as lung cancer [47]. The GWAS catalogue identified two SNPs in *LATS1* (rs17080102, rs62434129) that are associated with SBP. Additionally, rs62434129 has been linked with DBP and MAP [48].

For the most significant pathway identified by GSEA, the *GHRL* gene was involved. *GHRL* encodes the ghrelin-obestatin preprotein. This results in two peptides after cleavage: ghrelin and obestatin. Ghrelin is a pleiotropic hormone produced by enteroendocrine cells in the gastrointestinal tract. Of its many actions, its role as an appetite hormone is well recognised [49,50] and this is achieved through the activation of neuropeptide Y neurons in the anterior pituitary gland and hypothalamus [51]. In excess, ghrelin may cause hyperphagia and obesity [52]. In contrast, obestatin is an appetite suppressor [53]. Studies have consistently shown lower circulating levels of obestatin in patients with impaired glucose control, metabolic syndrome, T2D and obesity [54,55,56,57,58,59]. Therefore, our findings may suggest a potential role for hormone-mediated appetite regulation in the development of higher SBP in childhood.

While these findings point towards pathways with plausible links to cardiometabolic regulation, it is important to distinguish the associations observed in this cohort from mechanistic interpretations drawn from prior research. In this present study, *LATS1* and *GHRL* emerged as notable signals within exploratory analyses; however, the functional roles attributed to these genes largely reflect evidence from adult studies, GWAS associations and the broader biological literature rather than direct demonstration of childhood physiology. Our findings, therefore, provide hypotheses that warrant further investigation in larger, age-specific cohorts.

### 5.4. Limitations

Our main limitation in the infant cohort was the small sample size and substantial loss to follow-up. Despite this, significant relationships were established, thereby demonstrating the value in recruiting pregnant women at greater risk for FGR. Within the childhood cohort, we considered the potential influences of age, sex, ethnicity, height and growth trajectories on childhood SBP. While these factors did not meaningfully alter the observed patterns, the small sample size limits any adjustment and residual confounding cannot be ruled out. For both cohorts, while our inclusion criteria allowed us to assess relationships of SFG without necessarily resulting in SGA, generalizability may be limited if these mothers differ from the general population in ethnicity, socioeconomic status or health-seeking behaviours.

Metabolomic analysis was conducted by nuclear magnetic resonance, a fast, highly automatable, and reliable method [60]. Liquid chromatography–mass spectrometry and gas chromatography–mass spectrometry are alternative techniques that have been shown to have greater sensitivity for the detection of metabolites [60]. Therefore, it is possible that other relevant DEGs and DEMs exist, which have not been detected by this technique.

Lastly, whilst detection of ornithine from metabolomic analysis allowed identification of the arginine-NO pathway as a hypothesis-generating integrative model, with added confidence provided from detection on *ARG1* following transcriptomic analysis, epigenetic modifications may also play a role. This represents an important area for future work.

## 6. Conclusions

In both infancy and childhood, only a minority of those at higher risk of FGR were born SGA. Nevertheless, clear associations emerged between early life growth patterns and cardiometabolic risk markers—relationships consistent with those previously reported in cohorts of SGA with catch-up growth. These findings highlight the importance of postnatal surveillance for children exposed to SFG, regardless of their size at birth.

Pathway analyses suggested the presence of arginine-NO and phosphatidylinositol signalling pathways in adverse growth trajectories. Moreover, transcriptomic analyses alone identified a gene implicated in appetite regulation. These results raise the possibility that appetite-driven postnatal weight gain may contribute to elevated SBP in a subset of children with a specific early life transcriptomic signature. However, given the modest sample size and the exploratory nature of the analyses, these findings should be interpreted as candidate biological signals, which highlight pathways that warrant further investigation in larger cohorts to determine whether such molecular features could ultimately contribute to early life risk stratification.

## Data Availability

The datasets generated and analysed under the current study are available in the Gene Expression Omnibus (GEO) under accession number GSE312011. Additional supporting data are available from the corresponding author upon reasonable request.

## Abbreviations

The following abbreviations are used in this manuscript:

AC	abdominal circumference
AFP	alpha-fetoprotein
BMI	body mass index
∆child wt	weight SDS minus birthweight SDS)/age in years
DBP	diastolic blood pressure
DEGs	differentially expressed genes
DEMs	differentially expressed metabolites
FGR	fetal growth restriction
GSEA	gene set enrichment analysis
HC	head circumference
HDL	high density lipoprotein
LBW	low birthweight
LDL	low density lipoprotein
MAP	mean arterial pressure
MPC	Manchester Placenta Clinic
MUAC	mid-upper arm circumference
NO	nitric oxide
PAPP-A	pregnancy associated plasma protein A
PI	pulsatility index
RI	resistance index
SBP	systolic blood pressure
SGA	small for gestational age
SFG	suboptimal fetal growth
Sum SF	sum of skinfold thicknesses (infant cohort)
TC	thigh circumference
UtAD	uterine artery Doppler
∆fetal wt	birthweight centile minus 23 week centile estimated fetal weight), divided by days
